# Exploration of genome-wide DNA methylation profiles in night shift workers

**DOI:** 10.1080/15592294.2022.2152637

**Published:** 2022-12-01

**Authors:** Paul Wackers, Martijn E. T. Dollé, Conny T.M. van Oostrom, Linda W.M. van Kerkhof

**Affiliations:** Center for Health Protection, National Institute for Public Health and the Environment, Bilthoven, The Netherlands

**Keywords:** Night shift work, DNA-methylation, epigenetic clock, circadian rhythm

## Abstract

The past decades, studies indicated that night shift work is associated with adverse health effects, however, molecular mechanisms underlying these effects are poorly understood. A few previous studies have hypothesized a role for DNA-methylation (DNAm) in this relationship. We performed a cross-sectional epigenome-wide association study, to investigate if night shift work is associated with genome-wide DNAm changes and DNAm-based biological age acceleration, based on previously developed so-called ‘epigenetic clocks.’ Short term (2–6 years) and intermediate term (10–16 years) night shift workers, along with age and sex matched dayworkers (non-shift workers) were selected from the Lifelines Cohort Study. For genome-wide methylation analysis the Infinium Methylation EPIC array (Ilumina) was used. Linear regression analyses were used to detect differences in methylation at individual CpG-sites associated with night shift work. Pathway analysis was performed based on KEGG pathways and predictions of age acceleration in night shift workers were performed based on four previously developed epigenetic age calculators. Only in women, differences in methylation at individual CpG-sites were observed between night shift workers and non-shift workers. Most of these differentially methylated positions (DMPs) were observed in intermediate term night shift workers. Pathway analysis shows involvement of pathways related to circadian rhythm and cellular senescence. Increased age acceleration was observed only in short-term night shift workers (men and women). This might be indicative of adaptation to night shift work or a so-called healthy worker effect. In conclusion, these results show that DNA methylation changes are associated with night shift work, specifically in women.

## Introduction

The latest European Working Conditions Survey indicates that approximately 19% of the workers in the European Union (EU) work during the night at least once per month [1]. Night shift work is often defined as working at least 1 hour between midnight and 6.00 AM. Night (shift) work is associated with several long-term health effects including age-related diseases such as cardiovascular diseases and cancer [2–4]. Night shift work is known to disturb the circadian rhythms of many physiological processes in the human body [5,6]. It has been hypothesized that disturbance of these circadian rhythms underlies the health effects associated with night shift work [6,7]. Despite the great advancements in our understanding of the biological clock over the past decades, the molecular mechanisms underlying the health effects of shift work are still poorly understood. A better understanding of these mechanisms might aid in strategies to mitigate the associated negative health effects.

The past years, some studies have investigated DNA methylation as a possible mechanism through which chronic circadian disturbances might affect health [8–11]. Adams et al. hypothesized that since changes in DNA methylation are inducible by exogenous factors and changes in DNA methylation have been observed in the early stages of development of various chronic diseases, changes in DNA methylation might be occurring in night shift workers [8].

Recently, it was also suggested that shift work may result in increased age acceleration, since it has been associated with a higher risk for several age-related diseases [12,13]. The past years several methods to predict age-acceleration have been developed, some of which based on DNA methylation (DNAm) profiles [14]. These first so-called ‘epigenetic clocks’ were developed to predict chronological age. It was hypothesized that the residual variation may represent age-related biological variation and might provide an estimation of a person’s ‘biological age’ and thereby predict mortality risk and/or age-related disease risks. Subsequently, additional ‘epigenetics clocks’ have been developed and validated including aspects of several age-related clinical parameters [14,15].

In the current study, we performed a cross-sectional epigenome wide association study to investigate if night shift work is associated with genome-wide DNAm changes and DNAm-based age-acceleration in both male and female night shift workers. Night shift workers in this study were included if their night shifts comprised at least 3 hours between 00.00–05.00 and they worked at least 2 nights per month. These results will aid in further elucidating the biological mechanisms via which night shift work affects human health.

## Methods

### Study population and design

We used data from participants of the Lifelines Cohort Study. Lifelines is a multi-disciplinary prospective population-based cohort study examining, in a unique three-generation design, the health and health-related behaviours of 167,729 persons living in the north of the Netherlands. It employs a broad range of investigative procedures in assessing the biomedical, socio-demographic, behavioural, physical, and psychological factors which contribute to health and disease of the general population, with a special focus on multi-morbidity and complex genetics. The overall design and details on the methodology of the Lifelines cohort study can be found in previous papers [16,17].

Participants of the Lifelines Cohort study were recruited between 2007–2013 and were requested to visit one of the 12 Lifelines research facilities for a basic medical examination every 5 years. Participants were asked to fast overnight before blood draw between 8.00–10.00 a.m. during the visit at one of the Lifelines research facilities; 98% of our included participants complied (self-reported before blood draw). Blood was collected in EDTA-tubes and DNA was isolated from buffy coats with Qiagen Ultrapure by Lifelines Biobank upon our request (see below). DNA samples were stored by Lifelines Biobank at 4 ^◦^C.

Participants of the Lifelines cohort aged ≥18 years and with a valid email-address (n = 78,190) were approached in December 2017 or January 2018 with an additional digital questionnaire about their shift work history. These retrospective questions involved the participants’ shift work history and work schedule during the three months before blood samples were drawn in the second assessment round (2014–2017). The Lifelines study has been approved by the Medical Ethics Committee of the University Medical Centre Groningen, The Netherlands under number 2017/152. Written informed consent was obtained from all participants before entering the study [16].

### Data & sample collection

In total, 30,159 individuals (38.6%) responded to the shift work questionnaire. The questionnaire on shift work was designed to cover the major characteristics of shift work: current shift work status, frequency of night shifts (per month) and duration of night shift work in years [18,19]. Workers (> 12 hours per week) were defined as night shift workers in case they worked at least 3 hours between 00.00–05.00 [19], either in a rotating or permanent schedule. Night shift workers were included if they worked at least 2 nights per month during the three months before blood draw. Non-night shift workers were those participants who never performed shift work and had regular work times during the three months prior to blood draw (starting work after 06.00 and finishing work before 21.00). Participants were only included if a buffycoat sample was available.

**Figure 1.**
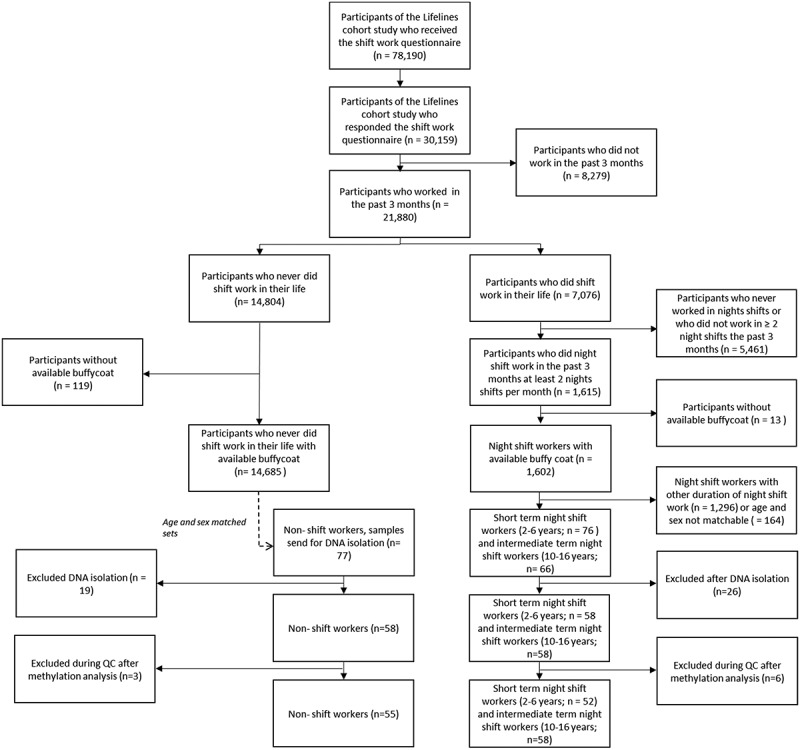
Flowchart of the study population: inclusions and exclusion.

Two groups of night shift workers were included: short-term shift workers (**short night SW**), who worked in night shifts for 2–6 years and intermediate term night shift workers (**int. night SW**), who worked in night shifts for 10–16 year. Age (±5 years) and sex randomly matched pairs were drawn between the short term and intermediate term night shift workers. Subsequently, these pairs of night shift workers were matched for age and sex with non-shift workers (**non-SW**), randomly out of 14,685 available non-shift workers. For several age ranges multiple possible matches were available between the groups, these samples were included as spares for DNA isolations. In total, 219 buffycoat samples were requested to be isolated for DNA. Samples were excluded after DNA isolation if the amount of DNA was insufficient (<1.5 µg), 260/280 ratio was not between 1.7–2.1, or when samples could no longer be appropriately matched for sex and age (± 5 years). This resulted in the inclusion of 174 samples (58 non shift workers, 58 short-term night shift workers and 58 intermediate term night shift workers) (Figure 1).

Of the 174 included samples, 500 ng of DNA was sent to NXT-DX, a research testing service for genome-wide methylation analysis using the Infinium Methylation EPIC array (HumanMethylation850 Bead Chip) by Ilumina in 2018, based on bisulphite conversion. Matched trio’s of samples were run on the same array, position on the array was randomized among groups and genders and blinded for the service provider.

### Data preparation

Handling, analysis, and visualization of the data were performed in R statistical software version 3.6.1 (http://cran.r-project.org). Raw signal intensities were imported in R using the minfi package [20] and subsequently subjected to quality control plots such as principal component analysis (PCA), density, – and sex-prediction plots. Based on these quality checks, one sample was excluded as a technical outlier. Eight samples were excluded based on discrepancy between reported sex and predicted sex. The PCA-plot as represented in S1 seemed to reveal one additional sample as a sex-outlier. However, the effect disappeared post-normalization. The final dataset comprised 165 samples for analysis (55 non-SW, 52 short night SW, 58 int. night SW).

The quality checks revealed batch effects caused by bisulphite conversion plate and hybridization slide. Per sex-group, stratified quantile normalization [21] was used to normalize the data using the standard Illumina probe design information (IlluminaHumanMethylationEPICanno.ilm10b4.hg19) [22]. Additional probe filtering was performed to remove from the dataset probes containing a SNP in the probe sequence and probes with a poor detection *p* value (*p* > 0.001). Normalized M-values were corrected for plate and slide effect using the combat function of the SVA package [23].

### Statistical methods

The Mann-Whitney U test or Independent samples T-test was applied to test for differences in main shift work characteristics between male and female night shift workers, and difference in body mass index (BMI) between non-shift workers and night shift workers. Heterogeneity in white blood cell composition was estimated using the Houseman method [24], the six white blood cell types were included in this estimation based on the reports of [25],and [26] [25,26]

Limma [27], a R package, was used to fit a linear regression to study the association between night shift work and individual probes. To account for heterogeneity, the regression model was adjusted for the estimated cell counts. A stringent p-value of p < 9*10^−8^ was used, as this was shown to adequately control for the false positive rate of EPIC array DNA methylation [28].

Pathway analysis was performed on DMP’s with a p < 9*10^−8^ (women) or p < 0.0005 (men) using the goregion function of the R-package missMethyl [29]. All probes measured in the specific contrast were used as background. Collection was set to KEGG, to test for overrepresented KEGG-pathways. Pathways with a False Discovery Rate (FDR) corrected p value < 0.05 were considered as significant.

Raw beta values were used to estimate the biological age, using four different epigenetic age calculators [15,30–32]. A t-test on age acceleration residuals was used to calculate the age acceleration difference between shift work groups. A p-value < 0.05 was considered significant.

## Results

### Description of the study population

In total, 165 participants were included after excluding nine samples during the quality control process. Mean age of the study population was 40.2 (standard deviation (SD) = 8.4) years old and 44.8% of them were men (Table 1). As participants were age-matched, no differences in age between groups were observed. The night shift workers worked on average 5.0 (SD = 2.0) night shifts per month with men working slightly more nights shifts per month on average (5.5; SD = 1.5) compared to women (4.6; SD = 2.3; p < 0.001). No significant differences in body mass index (BMI) were observed between any of the groups (Table 1).

**Table 1.** Characteristics of the study population.

Study population (n = 165)			
Women (n = 91)	**Non-SW (n = 31)**	**Short night SW (n = 28)**	**Int. night SW (n = 32)**
Age (in years, mean (SD))	40.1 (8.8)	37.9 (9.6)	39.5 (7.7)
BMI (in kg/M^2^, mean (SD)) (= 85)	24.1 (3.5)	26.6 (5.4)	25.0 (3.8)
Frequency of night shifts/ month (mean, SD)		4.2 (1.4)	4.9 (2.8)
Duration of night shifts in years (mean, SD)		3.7 (1.4)	13.9 (1.6)
Men (n = 74)	**Non-SW (n = 24)**	**Short night SW (n = 24)**	**Int. night SW (n = 26)**
Age (in years, mean (SD))	41.6 (8.3)	40.6 (7.9)	42.3 (8.1)
BMI (in kg/M^2^, mean (SD)) (n = 63)	25.3 (3.1)	25.7 (3.4)	27.2 (3.6)
Frequency of night shifts/ month (mean, SD)		5.4 (1.6)	5.5 (1.4)
Duration of night shifts in years (mean, SD)		4.1 (1.1)	13.9 (1.5)

Non-SW = non-shift workers, short night SW = short-term night shift workers (2–6 years), int. night SW = intermediate term night shift workers (10–16 years).

### Differentially methylated positions (DMPs) related to night shift work

A brief exploration of the dataset using a principal component analysis (PCA) showed that 11% of the variance in the dataset was explained by sex (Supplemental Fig. S1.). In line with more general views calling for sex stratified analyses [33] previously other researchers have shown that changes in methylation can differ between men and women [34,35]. In addition, we have previously (Streng et al) and in the current study (Table 1) observed that men and women differ in certain shift work characteristics. Therefore, in the current study, sex stratified analyses were performed. First, we performed linear regression analysis to investigate differences in methylation status at individual CpG sites (DMPs). Analyses were adjusted for estimated cell composition (Supplemental Fig. S2).

**Figure 2.**
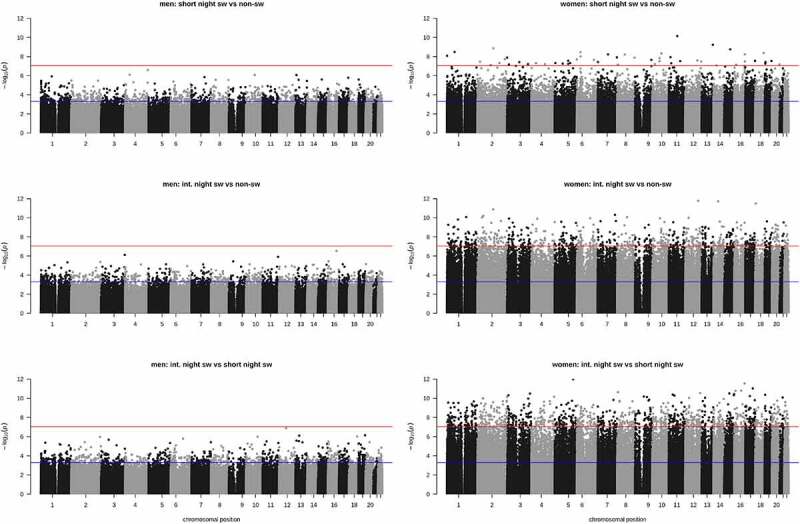
Manhattan plots depicting p-value for comparisons between groups in men (a) and women (b) per CpG site. Red line indicates p value of 9 *10^−8^ and blue line indicates p = 0.0005.

In men, no significant DMPs were observed when comparing the different groups, using a stringent p-value of p < 9*10^−8^ (Figure 2). When using a less stringent p-value of p < 0.0005, 1,259 and 845 positions were differentially methylated in short term night shift workers and intermediate term night shift workers, respectively, compared to non-night shift workers (Figure 2). When comparing short term night shift workers to intermediate term night shift workers 891 positions were differentially methylated (p < 0.0005). In women, with the stringent p-value < 9*10^−8^ as a threshold, 55 positions were differentially methylated in short-term night shift workers and 671 positions in intermediate term night shift workers compared to non-shift workers. When comparing short-term night shift workers to intermediate term night shift workers, 1,096 DMPS were observed (p < 9*10^−8^; Figure 2). A complete list of all DMPs is provided in the supplemental data. There was no overlap in the positions that were differentially methylated between groups in women (Supplemental Fig. S3).The number of DMPs in short term night shift workers that were hyper- or hypomethylated compared to non-shift workers was equal (49% hypermethylated). In intermediate term night shift workers more hypermethylation was observed (81% of DMPS) compared to non-shift workers and compared to short term night shift workers (85% hypermethylated). There was no overlap between the observed DMPs in women and the positions with lowest p-values observed in men (p value < 0.0005; Supplemental Fig. S4), nor did including age as a covariate in the linear regression analysis impact the results much, as expected since age-matching of subjects was applied (Supplemental Fig. S5). We have visualized the genomic locations of DMP’s in Supplemental Fig. S6.

Subsequently, we performed a pathway analysis to investigate if certain biological processes were particularly influenced by the differential methylation status related to night shift work in women. Two overrepresented pathways were observed (FDR <0.05) in the group of intermediate term female night shift workers. The pathway ‘cellular senescence’ is overrepresented in intermediate night shift workers compared to non-shift workers, with the significant genes in this pathway being LIN54,E2F1,ETS1,HIPK2,ITPR2,PIK3R1,RAD9A,CALM2, and MCU. The ‘circadian rhythm’ pathway is overrepresented in intermediate night shift workers compared to short-term night workers, with the significant genes in this pathway being CRY2, CSNK1D, PRKAA1, RORA and RORC. An overview of the results for the top 20 pathways in all groups is presented in the supplemental data.

### Epigenetic age clocks in night shift workers

**Figure 3.**
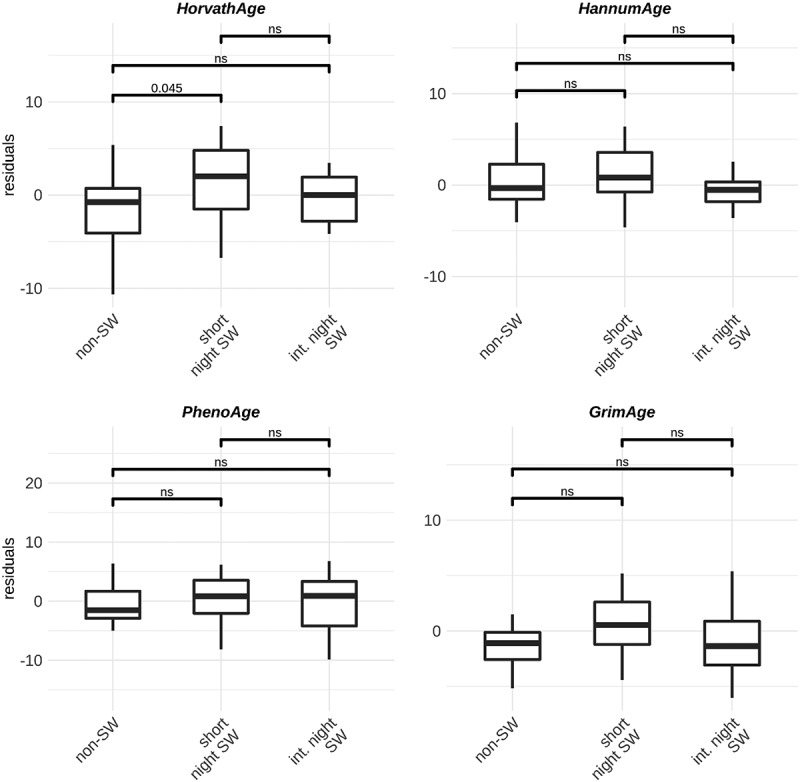
Boxplots of age acceleration residuals in **male** non-shift workers (n = 31), short term shift workers (n = 28) and intermediate term shift workers (n = 32) using four different models: Horvath Hannum, PhenoAge and GrimAge.

**Figure 4.**
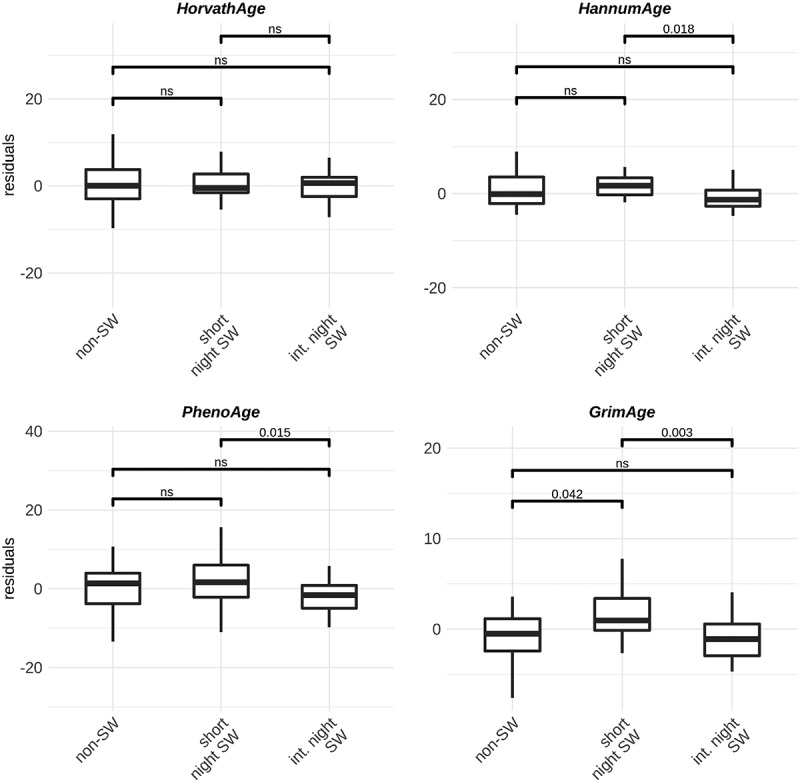
Boxplots of age acceleration residuals in **female** non-shift workers (n = 24), short term shift workers (n = 24) and intermediate term shift workers (n = 26) using four different models: Horvath Hannum, PhenoAge and GrimAge.

We investigated whether night shift work is associated with changes in epigenetic clocks as a predictor of biological age acceleration. We used four previously developed models: Horvath, Hannum, PhenoAge and GrimAge [15,30–32] and investigated the age acceleration residuals. In men, no differences between groups are observed when using the GrimAge, Hannum and PhenoAge clocks. However, using the Horvath clock, the model predicts slightly increased age acceleration in male short-term night shift workers compared to non-shift workers (Figure 3). In women, we observed increased age acceleration in short term shift workers compared to non-shift workers and intermediate term night shift workers when using the GrimAge clocks. When using the Hannum and PhenoAge clocks, we observed increased age acceleration in short term night shift workers compared to intermediate term night shift workers (Figure 4).

## Discussion

In this cross-sectional study, significant differences in genome-wide DNAm between night shift workers and non-shift workers were observed only in women. Most DMPs were observed in intermediate term night shift workers and these were mostly hypermethylated compared to non-shift workers. Pathway analysis shows involvement of pathways related to circadian rhythms and cellular senescence. On the other hand, increased age acceleration was observed only in short term night shift workers, in both men and women, depending on the age-calculator applied.

The observed difference between men and women with respect to DNA methylation patterns and the difference with respect to its association with night shift work has, to our knowledge, not been described before. Since there was no overlap in our study with the DMPs observed in female night shift workers and the positions with the lowest p-value in men (P < 0.0005), it is unlikely that this is related to the small difference in sample size between men and women in our study. Several previous studies have investigated genome-wide DNA methylation in night shift workers [Zhu, 8, 9, 13, 36]. However, all of these were among female (night shift) workers, exclusively. Considering our finding that no significant DMPs are observed in male night shift workers, there could potentially be a publication bias for studies among men. Alternatively, past study efforts may have had a primary focus on women considering the association of night shift work with breast cancer. The past decades it became more apparent that sex stratified analyses are needed to better understand health and disease [33] and as mentioned before, previously other researchers have shown that changes in methylation can differ between men and women [34,35]. Additionally, other factors that (potentially) differ between male and female night shift workers, such as lifestyle factors, could play a role. More studies, like ours, investigating DNA methylation in both sexes of night shift workers are needed to enhance our understanding of the role of sex in this relationship.

Two previously published studies investigating DNA methylation in female night shift workers reported DMPs associated with night shift work, with the highest number of significant DMPs observed in the study by Bhatti et al. (16,135 significant DMPs), albeit using a less stringent p-value compared to our study [Zhu, 9, 36]. Two studies reported no significant DMPs when comparing (night) shift workers with non-shift workers [8,13]. With respect to duration of night shift work, our finding that more changes in DNA methylation are present in night shift workers with longer duration of night shift work, is in line with the study by White et al. They observed 85 DMPs associated with years of night shift work, whereas none were associated with every shift work [13]. However in our study, DMPs in female intermediate night shift workers were mainly hypermethylated compared to non-shift workers or short-term night shift workers, whereas White et al. reported mainly hypomethylation of positions [13]. Somewhat similar to our findings Zhu et al reported slightly more hypermethylation (66%) among sites differentially methylated between shift workers and non-shift workers [Zhu, 36].

In the group of intermediate term night shift workers we observed changes in methylation related to two pathways: ‘circadian rhythm’ (compared to short term night shift workers) and ‘cellular senescence’ (compared to non-shift workers). This first pathway is in line with previous studies observing changes in methylation status of genes related to circadian rhythm in night shift workers [Zhu, 9{Ahmadi, 2022 #352{Ritonja, 2022 #355, 11, 13, 36]}}. Interestingly, in some of these studies the changes were also specifically observed in night shift workers with a longer history of night shift work [11,13] similar to our study. Additionally, there is some overlap of the involved genes compared to our study [Zhu, 37{Ritonja, 2022 #355, 9, 36]}. In our study, genes contributing to the overrepresentation of the circadian rhythm pathway were CRY2, CSNK1D, PRKAA1, RORA and RORC. For example, Zhu et al, using a targeted approach, observed hypermethylation of promoter regions of CRY2 [Zhu, 36] and Bhatti et al. observed changes at sites related to RORA, PRKAA1 and CSNK1D genes, among others that do not overlap with our study [9]. Interestingly, some previous studies using a targeted approach to investigate methylation status of circadian rhythm related genes, observed that changes among night shift workers in methylation status of these genes were related to other factors such as BMI and melatonin levels or were associated with sleep duration among day workers [37–39]. Most of the genes contributing to the overrepresentation of the circadian rhythm pathway in our study are so-called ‘core clock genes’ (CRY2, CSNK1D, RORA, RORC). The protein products of these genes are considered essential for the generation and regulation of circadian rhythms [40]. These results indicate that after longer periods of night shift work (10–16 years) epigenetic changes in processes related to the regulation of the circadian clock occur.

The second pathway overrepresented in intermediate term night shift workers compared to non-shift workers is ‘cellular senescence,’ with the significant genes in this pathway being LIN54, E2F1, ETS1, HIPK2, ITPR2, PIK3R1, RAD9A, CALM2, and MCU. Interestingly, ‘cellar senescence’ also came out forth among the top 20 enriched pathways comparing intermediate to short term night shift workers, albeit not significant after FDR correction (supplemental data), with four directly overlapping significant DMPs and associated/nearest genes. Moreover, all 18 DMPs associated with the cellular senescence pathway and significant in either or both comparisons showed similar directional changes using intermediate term night shift workers as reference (Supplemental Data). This indicates that the cellular senescence pathway is mainly affected in the intermediate term night shift group compared to the other two groups. Cellular senescence is known to be caused by DNA-damage and results in a senescence associated secretory phenotype comprising among others chemokines and interleukins, eventually disrupting the surrounding tissue and creating a cancer prone environment [41]. In this respect, our results might be in line with the DNA replication, recombination and repair pathway [Zhu, 9, 36] and the multiple immune [Zhu, 9, 36] pathways found enriched in two other DNA-methylation studies regarding night shift work, and the disease phenotypes night shift work is associated with [2–4].

We observed increased age acceleration in short term night shift workers (2–6 years), but not in intermediate term night shift workers (10–16 years), whereas more DNA methylation changes at CpG site level were present in the intermediate term night shift workers (for women). It is possible that this reflects some form of adaptation or it reflects a selection process, often referred to as the healthy (shift) worker effect [42]. Night shift workers who have difficulties coping with night shift work are more likely to switch to a non-shift working job. As we observed healthier metabolic profiles in night shift workers after more than 20 years of working in night shifts in two previous studies [43,44], in the current study we decided to investigate intermediate term night shift workers instead of long term night shift workers (> 16 years). However, it cannot be ruled out that a healthy worker effect is already present in this intermediate term (10–16 years of night shift work) group.

Our findings with the epigenetic clocks are not in line with previously published results by White et al. They reported an association between years of night shift work and age acceleration as predicted by epigenetic clocks (Horvath, Hannum and PhenoAge) in women. In their study no association was observed in women who worked in shifts for less than 10 years. Carugno et al. investigated age acceleration (using a predictor developed by Zbiec-Piekarska) among nurses and observed associations with night shift work only in specific subgroups: night shift workers with overweight/obesity and those experiencing work-related stress [12]. Differences in the included study population with respect to for example BMI and job-related factors might possibly explain these differences among study results. In the current study, we were unable to include potential confounders or mediators such as BMI, smoking, alcohol use, education level and working in night shift just prior to blood draw. Data on some of these variables is available within the Lifelines cohort study, however, due to missing data on these variables sample size in our current study was insufficient to include these factors into the analysis. Likewise, the limited sample size did not allow for more in depth analyses, for example with respect to intensity of night shift work (i.e., number of nights per month or number of consecutive night shifts) . More studies in different type of study populations, in particular in men, are needed to further understand the relationship between night shift work, DNA methylation and biological age acceleration and further validate our findings.

Another limitation of the current study is that it is not directly clear how the changes in methylation relate to functional effects. We investigated epigenetics changes in blood of night shift workers, which might not reflect the epigenetic signature in other tissues that are involved in increased health risks observed in night shift workers. However, white blood cells, being part of the immune system, are directly involved in some of the disease endpoints associated with night shift work and have been observed to be altered in night shift workers [44,45]. Examples are arterial plaque formation in the realm of cardiovascular disease and increased infection susceptibility among night shift workers [3,46,47].

To summarize, our results indicate that changes in DNA methylation in night shift workers differ between men and women. Most changes at DMP level were observed in female intermediate term night shift workers, whereas predicted increased age acceleration was observed in both male and female short term night shift workers. Involved pathways are related to circadian rhythm and cellular senescence, which may be pointing towards a potential link of disturbance of the biological clock and cancer related processes. Further studies are needed to better understand the relationship between nights shift work, DNA methylation and associated disease risks.

## Supplementary Material

Supplemental Material

## Data Availability

Data used in this study were obtained from or collected by the Lifelines Cohort Study. Data is available upon request according to their permissions for use. Please see www.lifelines.nl/researcher.

## Protection of research participants

Approval of the Lifelines Cohort study was obtained from the Medical Ethics Committee of the University Medical Center Groningen, The Netherlands. Informed consent was obtained from all participants.
