# Supplementary material for: Exploration of genome-wide DNA methylation profiles in night shift workers
Source: Epigenetics. 2022 Dec 1;18(1):2152637. doi: 10.1080/15592294.2022.2152637 (PMC9980630; doi:10.1080/15592294.2022.2152637)
Supplement: Supplemental Material [file KEPI_A_2152637_SM2215.zip › supplement/Supplemental Figures Wackers et al.docx]

**Supplemental Figures Wackers et al.**

**
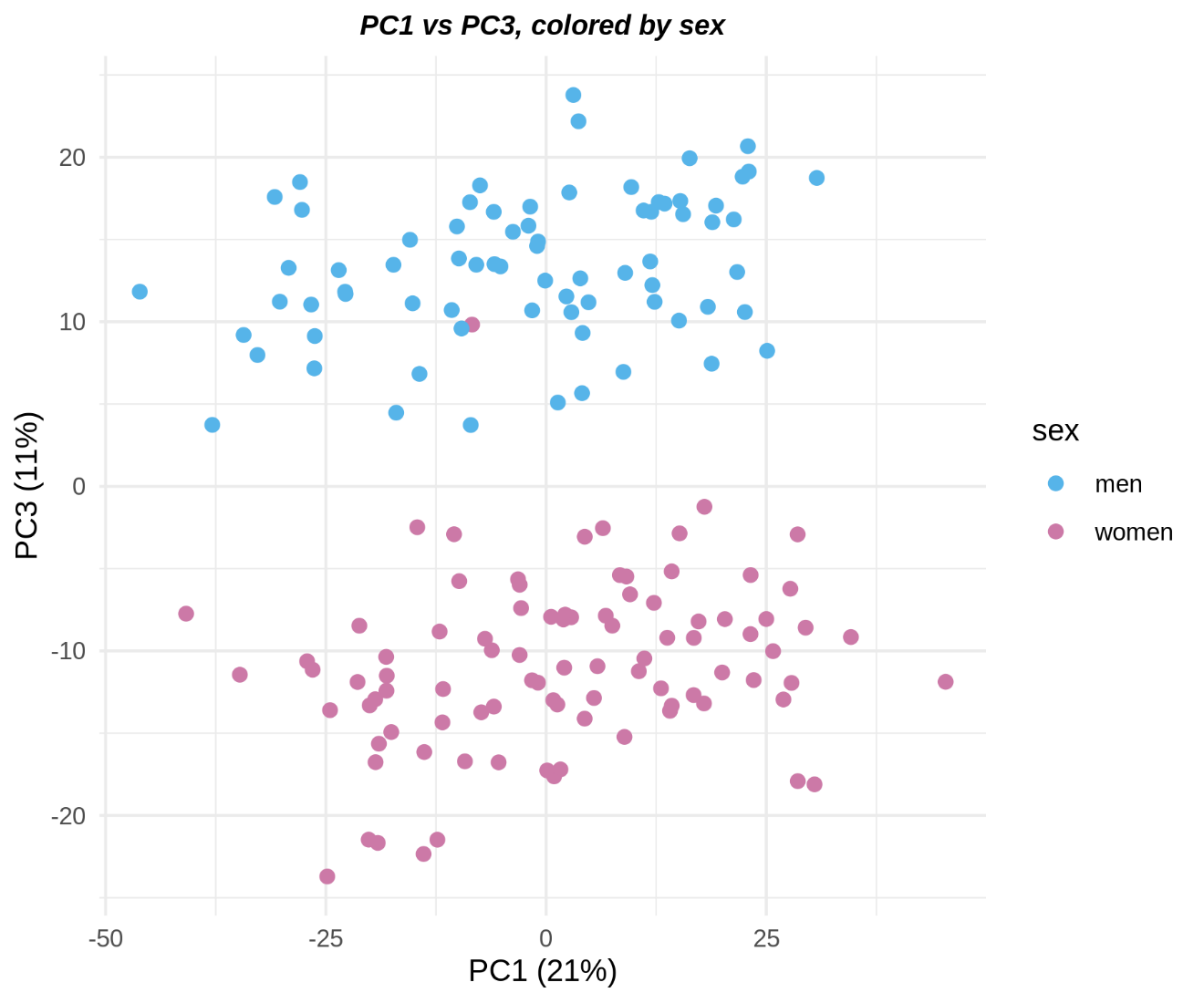
**

**Supplemental Figure S1** Plot of PCA analysis on pre-normalized data with PC 1 vs PC3 indicating proportion of variance by sex.


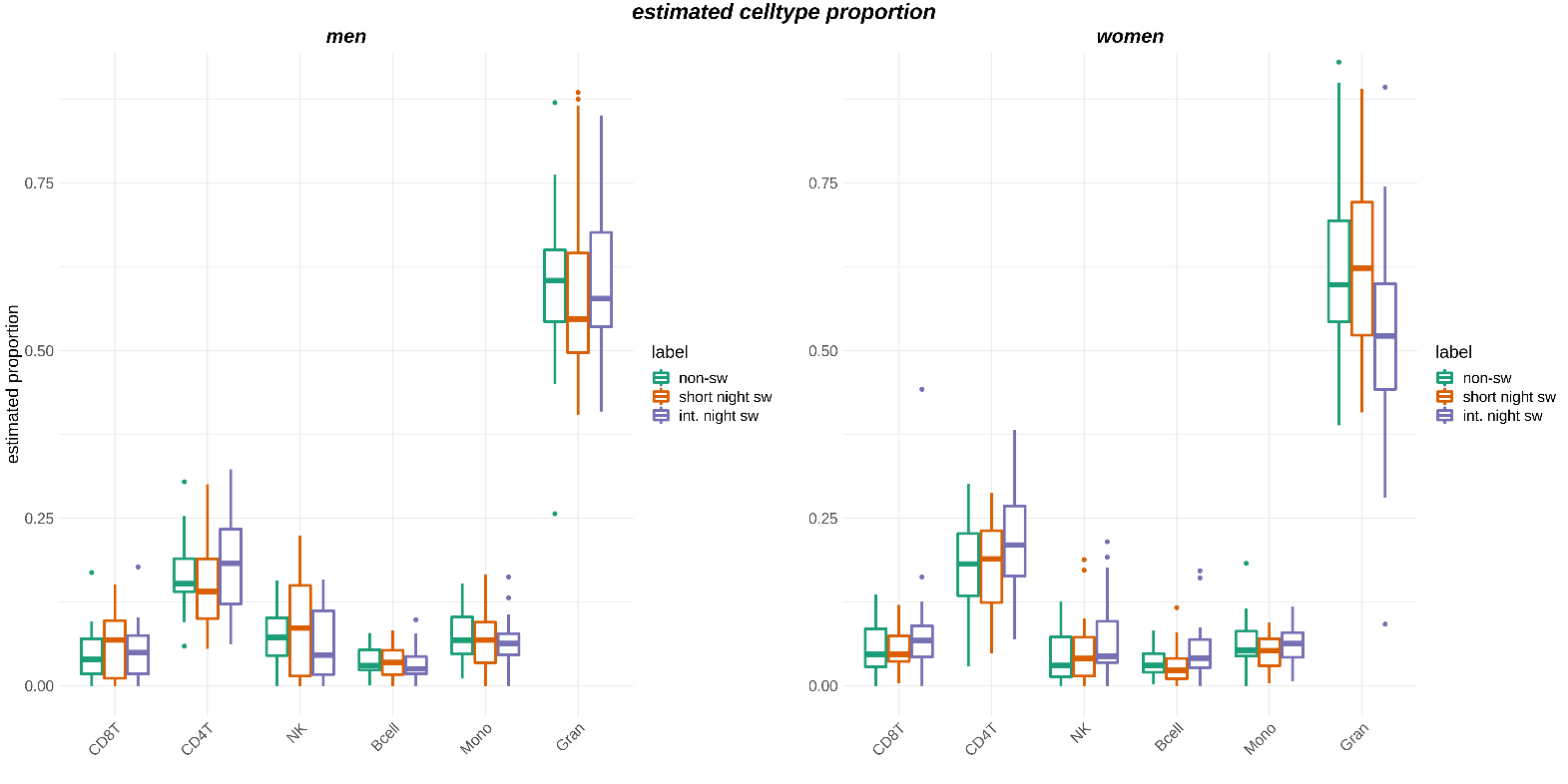


**Supplemental Figure S2** Estimated proportion of cell types in women (A) and men (B). Proportions in non-shift workers (non-SW; green), short term night shift workers (short night SW; orange) and intermediate night shift workers (int. night SW; purple).

**
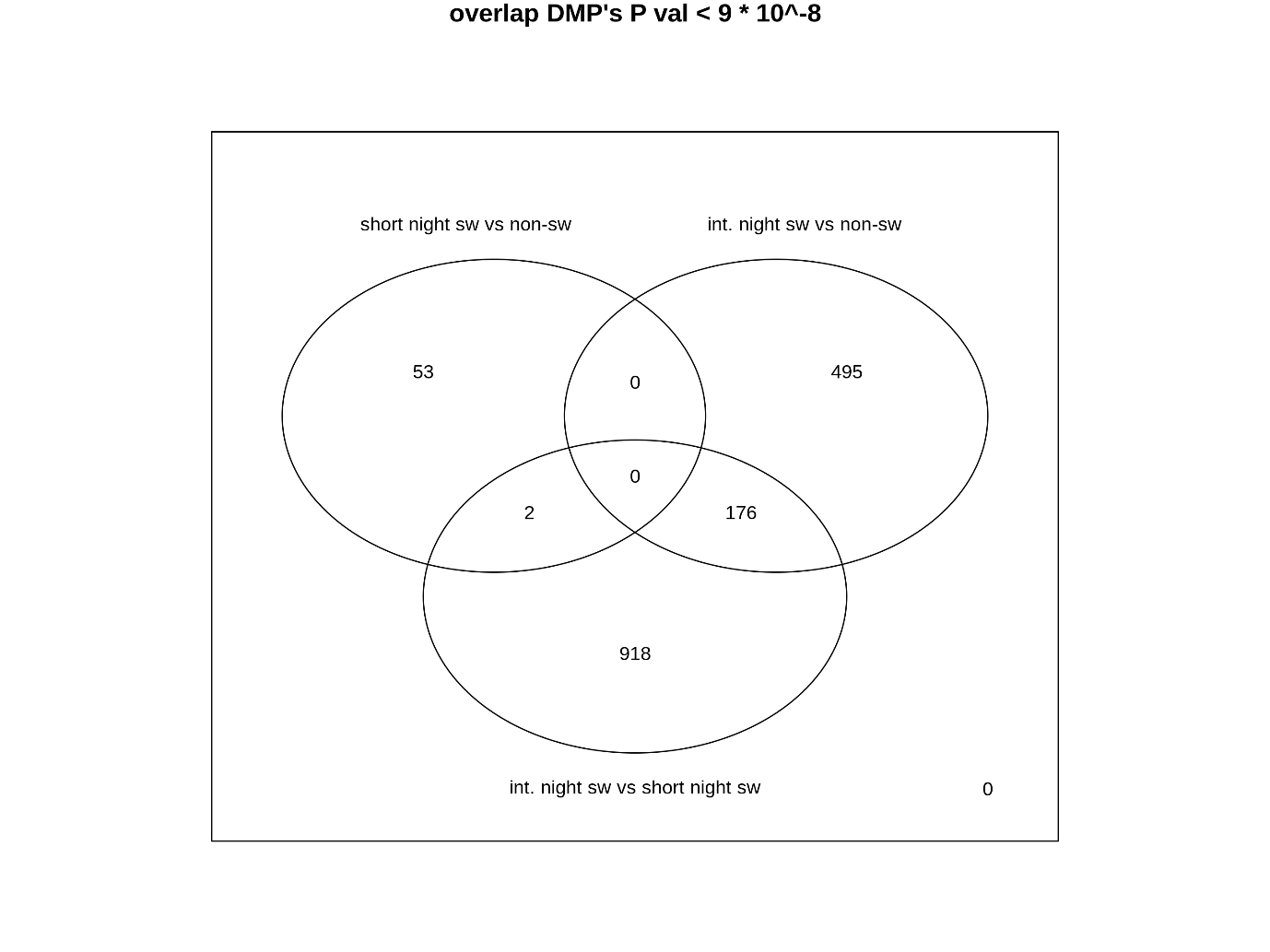
**

**Supplemental Figure S3** Overlap of differentially methylated positions in different **female** subgroups (p < 9*10^-8^). Non-SW = non- shift workers (n=31), short night SW = short term night shift workers (n= 28), int. night SW = intermediate term night shift workers (n = 32).

**
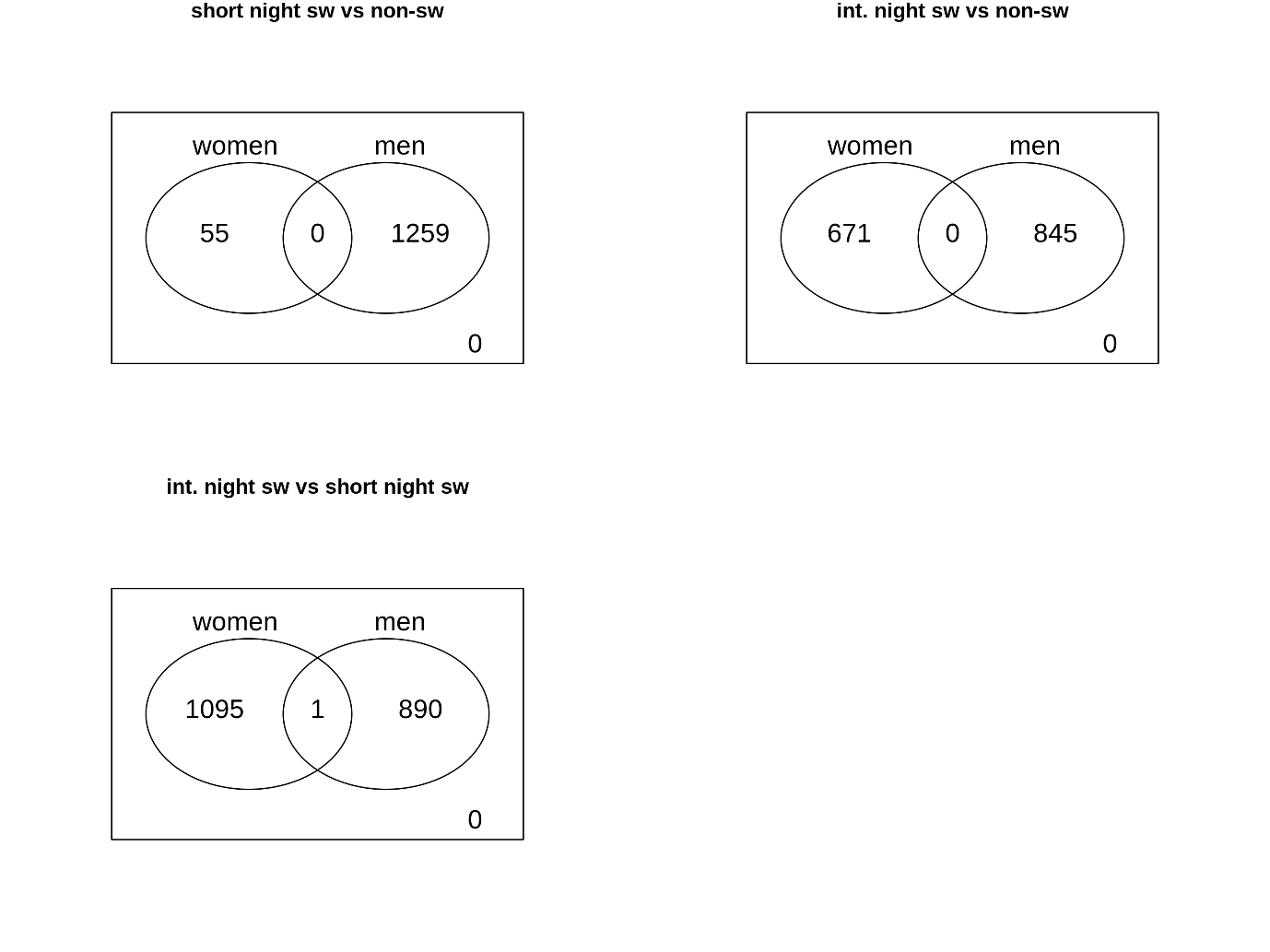
**

**Supplemental Figure S4** Venn diagram to visualize the overlap of differentially methylated positions between men and women. For women DMP’s with a p value < 9*10-8 are included. For men DMP’s with a p value < 0.0005.


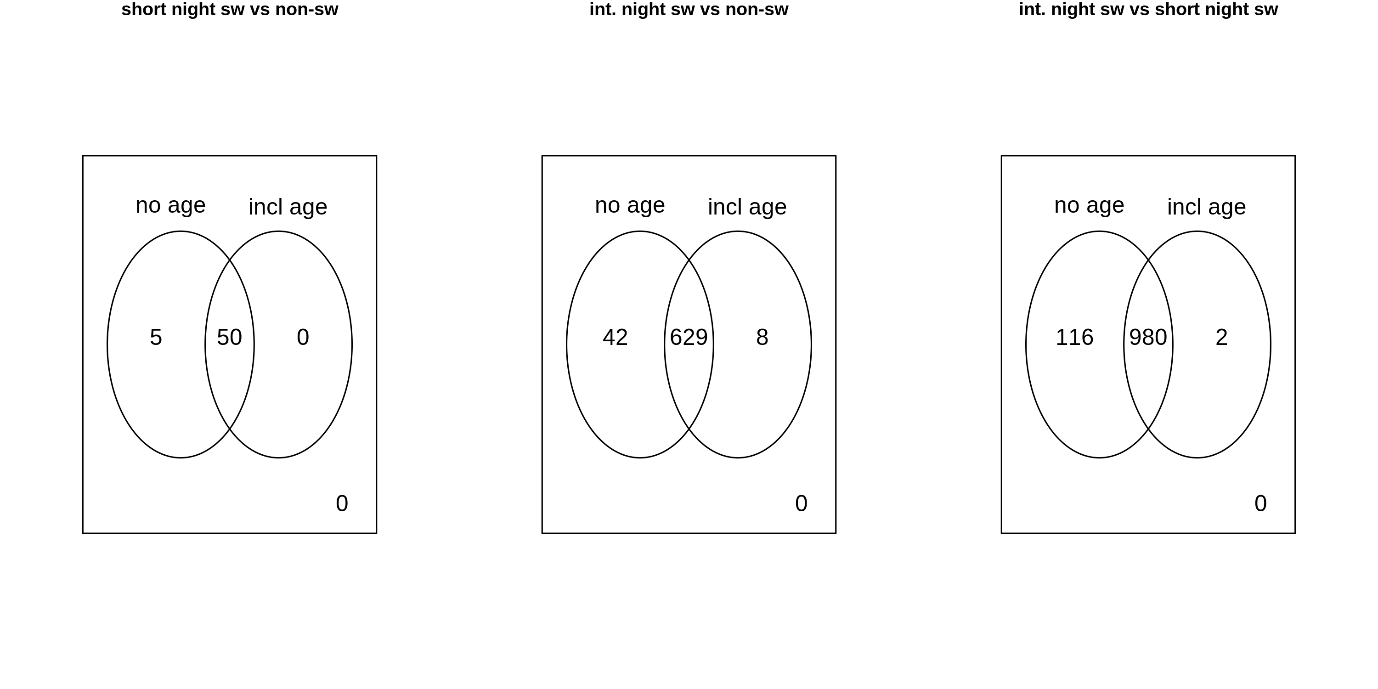


**Supplemental Figure S5** Venn diagrams to visualize the overlapping DMP's (p value < 9E-8) between the linear models including and excluding age in women. No DMP's (p value < 9E-8) were found in men including as well excluding age in the linear model.


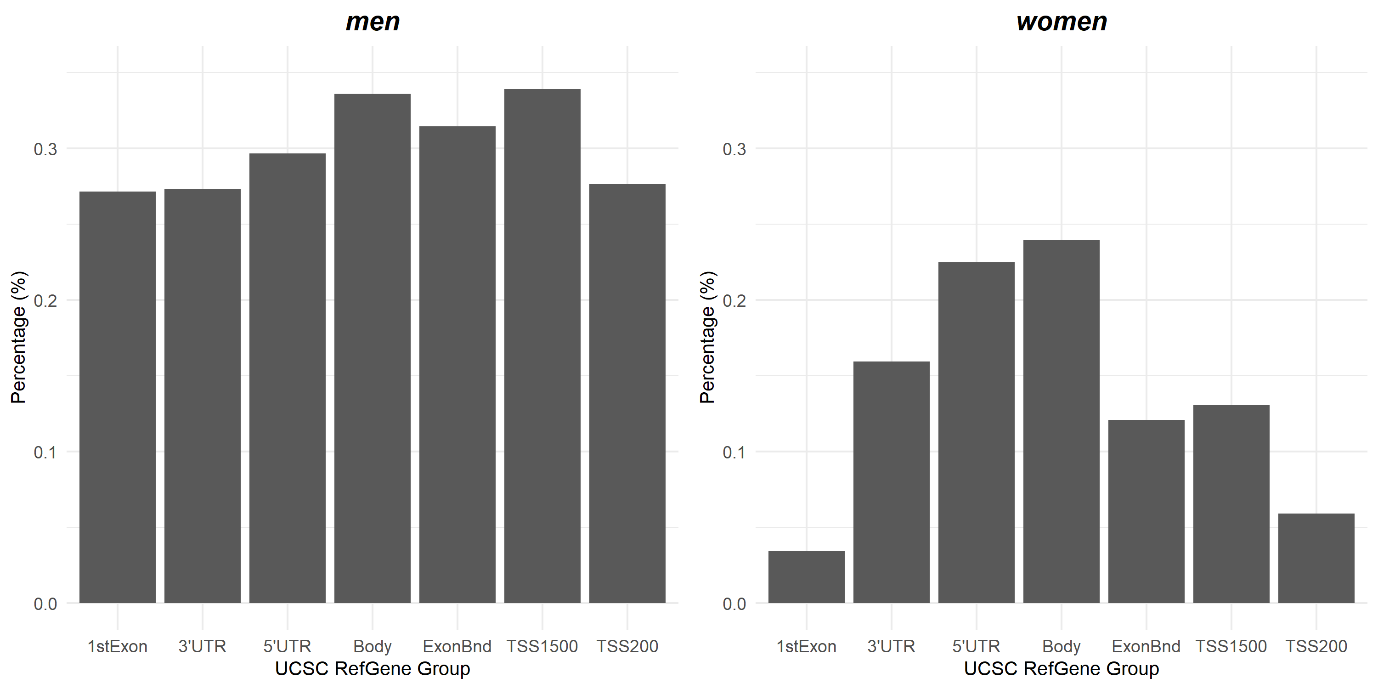


**Supplemental Figure S6** Barplot to visualize the distribution of genomic regions (based on UCSC regfene group) of the DMP's in men (p value < 0.0005) and women (p value < 9E-8). The percentages on y-axis are with respect to the total number of occurences of each group in the annotation of the array.

**Supplemental Figures Captions**

**Supplemental Figure S1** Plot of PCA analysis on pre-normalized data with PC 1 vs PC3 indicating proportion of variance by sex.

**Supplemental Figure S2** Estimated proportion of cell types in women (A) and men (B). Proportions in non-shift workers (non-SW; green), short term night shift workers (short night SW; orange) and intermediate night shift workers (int. night SW; purple).

**Supplemental Figure S3** Overlap of differentially methylated positions in different **female** subgroups (p < 9*10^-8^). Non-SW = non- shift workers (n=31), short night SW = short term night shift workers (n= 28), int. night SW = intermediate term night shift workers (n = 32).

**Supplemental Figure S4** Venn diagram to visualize the overlap of differentially methylated positions between men and women. For women DMP’s with a p value < 9*10-8 are included. For men DMP’s with a p value < 0.0005.
